# Role and Regulation of Pro-survival BCL-2 Proteins in Multiple Myeloma

**DOI:** 10.3389/fonc.2018.00533

**Published:** 2018-11-20

**Authors:** Anne Slomp, Victor Peperzak

**Affiliations:** Laboratory of Translational Immunology, University Medical Center Utrecht, Utrecht University, Utrecht, Netherlands

**Keywords:** apoptosis, B-cell malignancy, BCL-2 family, BH3-mimetic, germinal center, MCL-1, multiple myeloma, plasma cell differentiation

## Abstract

Apoptosis plays a key role in protection against genomic instability and maintaining tissue homeostasis, and also shapes humoral immune responses. During generation of an antibody response, multiple rounds of B-cell expansion and selection take place in germinal centers (GC) before high antigen affinity memory B-cells and long-lived plasma cells (PC) are produced. These processes are tightly regulated by the intrinsic apoptosis pathway, and malignant transformation throughout and following the GC reaction is often characterized by apoptosis resistance. Expression of pro-survival BCL-2 family protein MCL-1 is essential for survival of malignant PC in multiple myeloma (MM). In addition, BCL-2 and BCL-XL contribute to apoptosis resistance. MCL-1, BCL-2, and BCL-XL expression is induced and maintained by signals from the bone marrow microenvironment, but overexpression can also result from genetic lesions. Since MM PC depend on these proteins for survival, inhibiting pro-survival BCL-2 proteins using novel and highly specific BH3-mimetic inhibitors is a promising strategy for treatment. This review addresses the role and regulation of pro-survival BCL-2 family proteins during healthy PC differentiation and in MM, as well as their potential as therapeutic targets.

## Introduction

Multiple myeloma (MM) is a malignancy of clonal long-lived plasma cells (PC) residing in the bone marrow (BM) (1). The malignancy arises as a result of genetic changes that occur during differentiation of B-cells into PC (2, 3). MM is characterized by resistance against the intrinsic apoptosis pathway, which is regulated by proteins of the BCL-2 family (4).

The BCL-2 protein family consists of pro-survival BCL-2-like proteins (BCL-2, BCL-B, BCL-W, BCL-XL, BFL-1/A1, and MCL-1), pro-apoptotic BH3-only proteins (initiators), and pro-apoptotic effectors BAX, BAK (5), and possibly BOK (6–8). Cytotoxic stimuli such as DNA damage, chemotherapeutic agents, or cytokine deprivation promote upregulation of BH3-only proteins, which inhibit pro-survival BCL-2 family members (5). In addition, post-translational modification of BH3-only proteins can affect their stability, activity, and subcellular localization (9). BH3-only proteins vary in their affinities for different pro-survival proteins. For instance, BAD only binds with high affinity to BCL-2, BCL-XL, and BCL-W, while NOXA selectively inhibits MCL-1 and BFL-1/A1. BIM, PUMA, and BID have high affinity for all pro-survival proteins (10, 11). If all available pro-survival proteins are sequestered by BH3-only proteins, BAX and BAK can disrupt the mitochondrial outer membrane, leading to cytochrome C release, caspase activation, and execution of apoptosis (12). In addition, some BH3-only proteins, including BIM, PUMA, and BID, can directly bind to BAX or BAK and induce conformational changes that contribute to BAX/BAK activation (13–15). Regulation of apoptosis is essential for generation and selection of high-affinity PC, and malignant transformation of cells in this process often coincides with defects in apoptosis.

## Healthy PC differentiation

Long-lived PC originate from germinal centers (GC), which are dynamic structures that develop in secondary lymphoid organs upon antigen stimulation and helper T-cell activation. Clonal expansion, somatic hypermutation, class switch recombination, and affinity-based selection of B-cells take place in GCs, resulting in the production of high-affinity antibodies (16). GCs contain a dark zone (DZ), consisting of dividing B-cells, and a light zone (LZ), in which B-cells are selected based on antigen affinity through B-cell receptor (BCR) signaling and CD40-CD40L interactions (17–19). B-cells with low antigen affinity undergo apoptosis, and B-cells with high antigen affinity either return to the DZ for another round of mutation and expansion, or differentiate and move out of the GC as memory B-cells or PC. Somatic hypermutation and class switch recombination take place during proliferation in the DZ and are mediated by activation-induced cytidine deaminase (AID) (20). Most GC-derived PC are recruited into the BM, where stromal cells provide signals for long-term survival (21, 22).

### The BCL-2 family in PC differentiation

**Figure 1:**
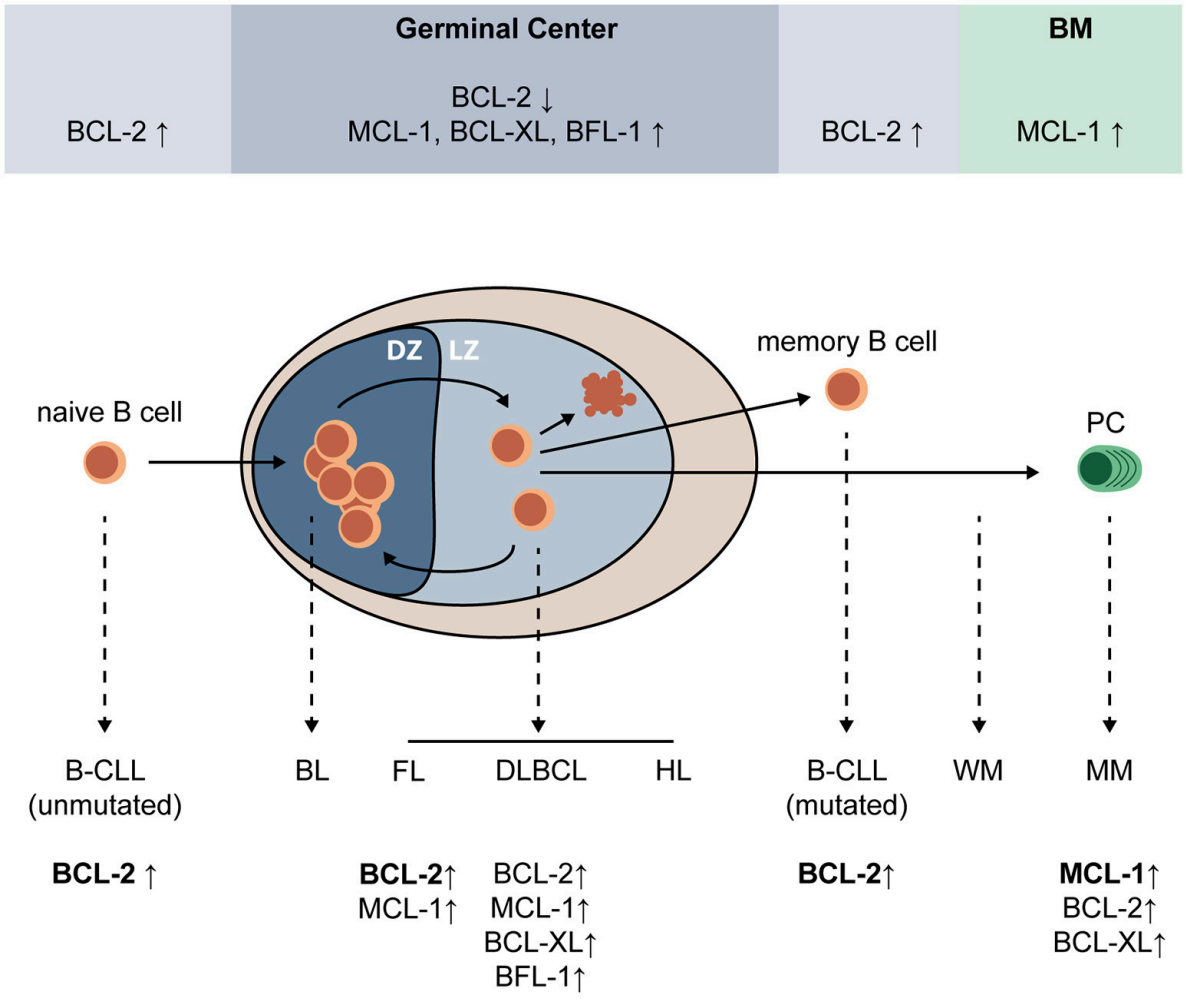
Expression of pro-survival BCL-2 family proteins during PC differentiation and after malignant transformation of (post-) GC B-cells. Upon encounter of a naïve B-cell with its cognate antigen, and in the presence of adequate T cell help, a germinal center (GC) is formed where the B-cell undergoes multiple cycles of expansion and hypermutation in the dark zone (DZ), and affinity-based selection in the light zone (LZ). Low-affinity B-cells undergo apoptosis, while high-affinity B-cells can undergo further selection, or exit the GC as a memory B-cell or plasma cell (PC). In the GC, BCL-2 expression is strongly repressed and expression of MCL-1, BCL-XL, and BFL-1 is increased. MCL-1, but not BCL-XL, was shown to be essential for survival of GC B-cells. Naïve and memory B-cells have high expression of BCL-2 and are sensitive to its inhibition, and PC depend on MCL-1 expression for survival. Erroneous targeting of activation-induced cytidine deaminase (AID) during somatic hypermutation and class switch recombination can lead to mutations that promote malignant transformation, resulting in a variety of GC-derived malignancies (dashed lines). Multiple GC-derived malignancies, such as follicular lymphoma (FL), diffuse-large B-cell lymphoma (DLBCL), some B-cell chronic lymphocytic leukemias (B-CLL), and multiple myeloma (MM) depend on overexpression of BCL-2 family proteins for survival. BL, Burkitt's lymphoma; BM, bone marrow; CLL, chronic lymphocytic leukemia; DLBCL, diffuse-large cell B-cell lymphoma; DZ, dark zone; FL, follicular lymphoma; HL, Hodgkin lymphoma; LZ, light zone; MCL, mantle cell lymphoma; MM, multiple myeloma; PC, plasma cell; WM, Waldenström macroglobulinemia.

Apoptosis regulation plays a central role in the cycle of expansion, selection, and differentiation that eventually produces mature PC. Expression of BCL-2 family proteins during PC differentiation and after malignant transformation of (post-) GC B-cells is highly variable and shown in Figure 1. MCL-1 is essential for GC formation and maintenance, memory B-cell development (23), and survival of existing PC (24). In fact, B-cells are dependent on MCL-1 throughout development (25). BCL-2, which is important for naïve and memory B-cells, is downregulated in the GC (26, 27). In contrast, MCL-1, BCL-XL, and BFL-1 are upregulated. BH3-only proteins BIM and BIK are also upregulated in the GC, but this upregulation was shown to be countered by MCL-1 and BCL-XL, respectively (27). Apoptosis of low-affinity B-cells in the GC is dependent on the interplay between pro-survival and pro-apoptotic BCL-2 proteins. In mice, overexpression of Bcl-2 was shown to disrupt GC selection of memory B-cells, but not of high-affinity plasmablasts (28). Knockout of Bim (29) or Noxa (30) resulted in increased amounts of low-affinity B-cells, suggesting that these BH3-only proteins play a critical role in elimination of low-affinity B-cell and PC clones. Puma was shown to be essential for regulation of memory formation in mice, since its loss resulted in accumulation of memory B-cells (31). Fully differentiated GC-derived PC are characterized by high expression of transcriptional regulator BLIMP-1, which promotes MCL-1 expression and represses BIM (32).

## Malignant transformation of GC B-cells

In the GC, somatic hypermutation and class switch recombination are mediated by AID, which functions by deaminating cytidine residues to uracil (20). AID is targeted to the variable immunoglobulin (Ig) regions, as well as the Ig switch regions. As a result of AID activity, the mutation rate in the variable Ig regions is estimated to increase to between 10^−2^ and 10^−3^ mutations per bp (33). In addition to its function in the Ig gene, AID can also be erroneously targeted to other genomic loci, introducing mutations and Ig translocations that can contribute to malignant transformation (34, 35). Many different malignancies, some of which dependent on BCL-2 family proteins for survival, arise from (post-) GC B-cells (Figure 1). These include B-cell chronic lymphocytic leukemia (CLL) (36), follicular lymphoma (37), diffuse-large B-cell lymphoma (DLBCL) (38), Waldenström macroglobulinemia (WM) (39), and multiple myeloma (MM) (40).

### Pro-survival BCL-2 proteins in GC B-cell malignancies

Pro-survival BCL-2 proteins contribute to apoptosis resistance of malignant B-cells, and their overexpression can be regulated in different ways. In 60–65% of CLL cases, the BCR is hypermutated, indicating that the malignancy originates from post-GC B-cells. Conversely, in the remaining 35–40% of cases, the BCR lacks signs of hypermutation and the disease presumably originates from B-cells that have differentiated independently of the GC (36). In both types, apoptosis resistance is mediated by overexpression of BCL-2 (41). This overexpression is due to *BCL2* gene hypomethylation and genetic loss of microRNA loci that normally inhibit BCL-2 expression (42, 43). Inhibition of BCL-2 using specific BH3-mimetic inhibitor Venetoclax efficiently induces apoptosis in CLL cells in circulation, and is also promising for other BCL-2 dependent malignancies such as follicular lymphoma and a subset of DLBCL (44–46).

Follicular lymphoma originates from GC B-cells and is characterized by the hallmark chromosomal translocation t(14;18), which is present in 85% of patients and results in overexpression of BCL-2 due to juxtaposition of the Ig heavy chain (*IGH*) and *BCL2* loci (37). In addition, MCL-1 is highly expressed in some follicular lymphomas, and its expression correlates with disease grade (47).

DLBCL has distinct subtypes, including germinal center B-cell-like (GCB-) DLBCL, which is derived from normal GC B-cells; and activated B-cell-like (ABC-) DLBCL, originating from B-cells that have completed the GC reaction (48). T(14;18) is present in 45% of GCB-DLBCL, but does not occur in ABC-DLBCL (49). Still, *BCL2* expression is high in many cases of ABC-DLBCL, as a result of gain or amplification of the 18q chromosome arm on which *BCL2* is located (50). MCL-1 expression is also frequently high in ABC-DLBCL and sometimes in GCB-DLBCL, possibly as a result of chromosomal amplification or transcriptional regulation (51). In addition, ABC-DLBCL is characterized by constitutively high NF-κB activity. Among the targets of NF-κB are BCL-XL, BFL-1/A1, and possibly BCL-2, whose high expression as a result of NF-κB signaling may contribute to apoptosis resistance in ABC-DLBCL (52–54).

MM and WM are malignancies that contain a clonal PC population residing in the bone marrow. Both are preceded by monoclonal gammopathy of undetermined significance (MGUS), which is characterized by presence of <10% clonal PC in the BM, presence of monoclonal Ig in the blood, and lack of clinical symptoms (55, 56). WM originates from post-GC B-cells that have undergone somatic hypermutation but did not undergo class switching, whereas MM originates from post-GC B-cells after class switching (39). As a result, the serum Ig in WM is of the IgM type, and IgH translocations do not occur (57). The cellular phenotype is mixed, ranging from B-cells to PC (58). Possibly, malignancy is acquired during the B-cell or plasmablast stage, with some malignant cells continuously differentiating into PC. MM, on the other hand, consists of fully differentiated PC and is characterized by frequent IgH translocations and genomic instability (59). MM cells most frequently produce IgG or IgA, although IgM or IgD have been observed in rare cases (60). In WM, pro-apoptotic and pro-survival BCL-2 family proteins are expressed at low levels similar to non-malignant B-cells and PC. It is therefore expected that WM will only be sensitive to BH3-mimetic drugs if these are combined with other treatments that increase pro-apoptotic protein levels and mitochondrial priming (61). In contrast, MM cells are highly dependent on BCL-2 family proteins for survival, with MCL-1 as the essential player (62, 63).

## The BCL-2 family in multiple myeloma

**Figure 2:**
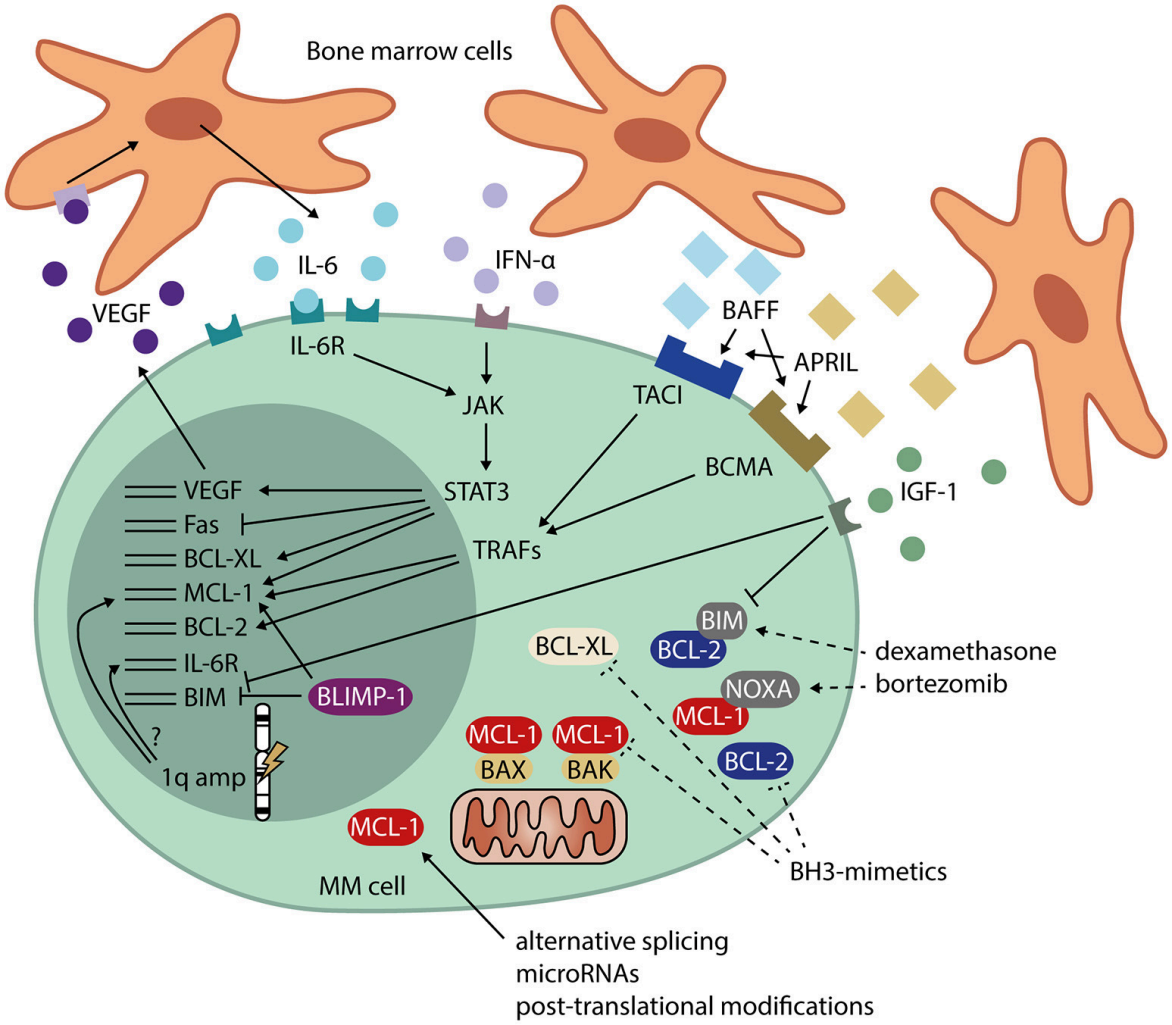
Signals and cellular processes that mediate apoptosis resistance in MM. MM cells receive signals from the bone marrow microenvironment that stimulate their survival. These signals include IL-6 and IFN-α, leading to JAK/STAT signaling and expression of MCL-1, BCL-XL, and VEGF. VEGF, in turn, promotes IL-6 production by neighboring cells. Other signals from the bone marrow microenvironment include BAFF and APRIL, which signal via TRAFs and induce expression of MCL-1 and BCL-2. IGF-1 signaling downregulates BIM, transcriptionally as well as post-translationally. MM cells also have high expression of the PC transcriptional regulator BLIMP-1, which promotes MCL-1 and represses BIM expression. Amplification of the 1q chromosome arm often occurs in MM. The genes for both MCL-1 and the IL-6 receptor (IL-6R) are present on this locus, possibly leading to overexpression in 1q-amplified MM. In addition to transcriptional regulation, MCL-1 is heavily regulated post-transcriptionally, which may contribute to the high MCL-1 protein levels found in MM. Dashed lines represent methods for interference in apoptosis resistance by MM drugs dexamethasone and bortezomib, and by BH3-mimetics. APRIL, a proliferation-inducing ligand; BAFF, B-cell activating factor; BCMA, B-cell maturation antigen; BH-3, BCL-2 homolog 3; BLIMP-1, B lymphocyte-induced maturation protein 1; IFN-α, interferon alpha; IGF-1, insulin-like growth factor 1; IL-6, interleukin 6; IL-6R, interleukin 6 receptor; JAK, janus kinase; STAT3, signal transducer and activator of transcription 3; TACI, transmembrane activator and calcium-modulating ligand interactor; TRAF, TNF receptor-associated factor; VEGF, vascular endothelial growth factor.

MCL-1 protein expression is increased in newly diagnosed MM compared to healthy PC, and protein levels are even higher at relapse (64). In addition, overexpression of MCL-1 is associated with shorter patient survival (64). Using RNA interference lethality screening in cell lines, MCL-1 was also identified as one of the most important and selective survival genes for MM (65). In subsets of MM cell lines and patient samples, BCL-2 and BCL-XL expression is also high (66), suggesting that these three proteins may act redundantly in preventing apoptosis. Since expression of both pro-survival and pro-apoptotic BCL-2 family members is heterogeneous, and the interplay between them is complex and dynamic, dependence on MCL-1, BCL-2, and BCL-XL is likely to differ between patients (66–68). Signals and cellular processes that may lead to overexpression of MCL-1, BCL-2, and BCL-XL in MM are indicated in Figure 2.

### Survival signals from the BM microenvironment

MM cells reside in the BM, where they interact with extracellular matrix proteins and cells from the BM microenvironment, which include stromal cells, osteoblasts, osteoclasts, endothelial cells, fibroblasts, adipocytes, and cells of hematopoietic origin (40). MM cells promote neighboring cells to produce IL-6 (69), which induces JAK/STAT3 signaling in MM, leading to transcription of MCL-1 and BCL-XL (70–73). MCL-1 expression in MM can also be IL-6-independent (74), or occur via other signals from the BM microenvironment (75). For instance, signaling through BAFF (B-cell activating factor) and APRIL (a proliferation-inducing ligand), whose levels are increased in MM patients compared to healthy controls, induces expression of both MCL-1 and BCL-2 and promotes PC survival (24, 76). Other survival signals from the bone marrow environment include interferon α (IFN-α), which induces MCL-1 in a STAT3-dependent manner (77), and insulin-like growth factor 1 (IGF-1), which downregulates expression of BIM (78).

### Genetic lesions

MM is characterized by recurrent chromosomal aberrations, some of which may be linked to apoptosis pathways. Translocations or chromosomal amplifications and gains involving 18q are rare in MM (79), suggesting that *BCL2* overexpression is not a key event in malignant transformation. No other genetic lesions in MM have directly been correlated to overexpression of a BCL-2 family member. Nevertheless, gain or amplification of 1q21, the chromosome region containing the *MCL1* gene, occurs in approximately 40% of MM cases and correlates with poor disease prognosis (80). Notably, *IL6R*, the gene encoding the IL-6 receptor, is also located on 1q21, as are several other candidate drivers of high-risk disease (81).

T(4;14), which is present in 10–15% of MM patients (80), may lead to disruption and subsequent overexpression of fibroblast growth receptor 3 (*FGFR3)*, which is considered an oncogene (79). In a murine IL-6-dependent hybridoma cell line, FGFR3 was shown to signal through STAT3 and substitute IL-6 signaling, leading to increased BCL-XL expression and decreased apoptosis (82). Correspondingly, specific tyrosine kinase inhibitors with known anti-FGFR3 activity induced apoptosis in t(4;14)-positive cell lines (83).

### MCL-1 stabilization

Unlike for BCL-2 and BCL-XL (66), transcriptional activity of *MCL1* does not directly correlate to protein levels. MCL-1 is unique within the BCL-2 family because it has a large N-terminal domain that allows for post-translational modification (84, 85). Proteasomal degradation of MCL-1 occurs upon phosphorylation and subsequent poly-ubiquitination of this N-terminal region. Kinases associated with phosphorylation of MCL-1 include JNK, GSK-3, and ERK-1 (86). Ubiquitin ligases Mule, SCF^β−TrCP^, SCF^Fbw7^, and APC/C^Cdc20^ were shown to target MCL-1 for proteasomal degradation after recognizing specific phosphorylated residues (87). This process can be reversed by deubiquitinases, such as USP9X (88). The contribution of these kinases and ubiquitin modifiers to MCL-1 regulation in MM is currently unknown. If the key players in MCL-1 regulation can be identified for MM, these MCL-1-modifying proteins may be interesting targets for therapeutic intervention.

## Overcoming apoptosis resistance: BCL-2 proteins as therapeutic targets in MM

As apoptosis resistance in B-cell malignancies often results from overexpression of pro-survival BCL-2 family proteins, inhibiting these proteins is a promising strategy for development of targeted therapeutics. Several BCL-2 family inhibitors, also named BH3-mimetics because of their structural and functional resemblance to the BH3 domain of BH3-only proteins, are currently in clinical development. BCL-2 inhibitor Venetoclax is the first BH3-mimetic approved by the Food and Drug Administration. It was approved in 2016 for treatment of CLL with a 17p deletion (46). Additionally, Venetoclax was tested in phase I clinical trials with relapsed and refractory MM patients, where monotherapy was particularly effective when the t(11;14) translocation was present (89). T(11;14) is associated with an increased *BCL2*/*MCL1* mRNA ratio, but the mechanism behind this is unknown (90). When MM patients were treated with Venetoclax in combination with conventional MM drugs bortezomib (a proteasome inhibitor) and dexamethasone, it was well tolerated and the response rate was highest in patients with high *BCL2* expression (91). Experiments in cell lines even indicate more-than-additive effects when Venetoclax is combined with proteasome inhibitor carfilzomib or dexamethasone, due to upregulation of NOXA and BIM, respectively (92). If conventional treatment increases availability of BH3-only proteins and their distribution toward pro-survival target proteins, this may increase sensitivity to BH3-mimetic drugs.

While the results of MM treatment with Venetoclax underline the potential of using BH3-mimetics in MM, they also suggest that Venetoclax may only be effective in a subset of patients, namely those who have relatively high BCL-2 and relatively low MCL-1. Based on *in vitro* and xenograft experiments, MCL-1 is often shown to be essential for MM survival and its generally high expression may confer resistance to Venetoclax (66, 93, 94). Therefore, MCL-1 itself is a very promising therapeutic target in MM, and multiple MCL-1 inhibitors are currently under development (95). MCL-1 inhibitor S63845 efficiently kills MM and other MCL-1-dependent cancer cell lines (96). Its derivate S64315/MIK665 is currently being tested in phase I clinical trials by Servier for acute myeloid leukemia and myelodysplastic syndrome (NCT02979366), and by Novartis for MM and DLBCL (NCT02992483). In addition, clinical testing in MM patients has started with MCL-1 inhibitors developed by Amgen, named AMG 176 and AMG 397 (NCT02675452 and NCT03465540, respectively) (97), and by AstraZeneca, named AZD5991 (NCT03218683) (98).

Simultaneous targeting of multiple BCL-2 family proteins may be a solution to resistance in case of redundancy between MCL-1, BCL-2, and BCL-XL in MM. Before the development of Venetoclax, BH3-mimetics with broader protein specificity have been studied, such as Navitoclax (99). Navitoclax (ABT-263) mimics the selectivity of BAD, thereby inhibiting only BCL-2, BCL-XL, and BCL-W. When tested in CLL patients, results were promising, but dose-limiting thrombocytopenia was observed as a result of BCL-XL inhibition (100–102). This led to the development of BCL-2-selective BH3-mimetic Venetoclax (44). Other putative BCL-2 family inhibitors with broad target specificity, such as Obatoclax (GX15-070), were shown to function partly or completely in a BAX/BAK-independent manner, and are therefore no longer considered BH3-mimetics (103). The results with Navitoclax indicate that potential side-effects of BCL-2 family inhibitors may be dose-limiting, and that combined inhibition of BCL-2 family members may only be possible if the concentration of each specific inhibitor remains below the threshold of toxicity.

MCL-1 is not only essential for B-cells and PC, it is also essential in other cell types, including hematopoietic stem cells (104), cardiomyocytes (105), and neural precursor cells (106). In contrast to healthy cells, increased expression of pro-apoptotic molecules (“priming”) renders malignant cells more susceptible to apoptosis upon inactivation of pro-survival proteins (107). Since MCL-1 is the most dominant pro-survival protein in MM, its inhibition leads to release of a large proportion of pro-apoptotic proteins present in MM cells, thereby promoting apoptosis induction. In mice, MCL-1 inhibitor S63845 was tolerated well at concentrations that killed cancer cells (96), even when murine Mcl-1 was replaced by its human ortholog, thereby increasing inhibitor sensitivity of all cells (108). This may yield a therapeutic window for targeting MCL-1, especially if MCL-1 inhibitors are combined with existing treatments that increase pro-apoptotic protein expression.

## Conclusion

High expression of pro-survival BCL-2 family proteins contributes to outgrowth and drug resistance of malignant B-cell clones. While beneficial for cell survival, addiction to high levels of specific pro-survival BCL-2 proteins also makes cells vulnerable to BCL-2 family inhibition using BH3-mimetic drugs. MM is characterized by high expression of MCL-1, and overexpression of BCL-2 and BCL-XL is observed in subsets of patients. Constitutive overexpression of these pro-survival proteins in MM results from a range of microenvironmental signals and different genetic lesions. This complex regulation of MCL-1, BCL-2, and BCL-XL offers multiple direct and indirect targets for therapeutic intervention. Recent development of BH3-mimetic drugs, that specifically target MCL-1, BCL-2, or BCL-XL, may contribute to overcoming apoptosis resistance and improving treatment for MM.

## Author contributions

AS and VP wrote the manuscript and designed the figures. Both authors read and approved the final manuscript.

### Conflict of interest statement

The authors declare that the research was conducted in the absence of any commercial or financial relationships that could be construed as a potential conflict of interest.

## Abbreviations

ABC-DLBCL: activated B-cell-like diffuse-large B-cell lymphoma
AID: activation-induced cytidine deaminase
APRIL: a proliferation-inducing ligand
BAFF: B-cell activating factor
BCL-2: B-cell lymphoma 2
BCR: B-cell receptor
BH3: BCL-2 homology 3
BM: bone marrow
CLL: (B-cell) chronic lymphocytic leukemia
DLBCL: diffuse-large B-cell lymphoma
DZ: dark zone
FGFR3: fibroblast growth factor receptor 3
GC: germinal center
GCB-DLBCL: germinal center B-cell-like diffuse-large B-cell lymphoma
IFN-α: interferon α
Ig: immunoglobulin
IGF-1: insulin-like growth factor 1
IgH: immunoglobulin heavy chain
IL-6: interleukin 6
LZ: light zone
MCL-1: myeloid cell leukemia 1
MGUS: monoclonal gammopathy of undetermined significance
MM: multiple myeloma
PC: plasma cell
WM: Waldenström macroglobulinemia.
